# How Efficient Are Monte Carlo Calculations Together With the Q-System to Determine Radioactive Transport Limits? Case Study on Medical Radionuclides

**DOI:** 10.3389/fmed.2022.675009

**Published:** 2022-07-13

**Authors:** Maddalena Maietta, Ferid Haddad, Sebastien Avila

**Affiliations:** ^1^Laboratoire Subatech, UMR 6457, IMT Nantes Atlantique, CNRS-IN2P3, Université de Nantes, Nantes, France; ^2^ARRONAX GIP, Nantes, France; ^3^Naogen Pharma, Nantes, France

**Keywords:** radioactive transport, radiopharmaceuticals, Monte Carlo, Q-system, container

## Abstract

The development of the so-called theranostics approach, in which imaging information are used to define a personalized therapeutic strategy, is driving the increasing use of radionuclides in nuclear medicine. They are artificially produced either in nuclear reactors, charged particle accelerators, or using radionuclide generators. Each method leads to radioisotopes with different characteristics and then clinical utility. In the first two cases they are extracted from stable or radioactive target bombarded with a particle beam. After extraction/purification of the target, the radionuclides, either implanted on solid or in liquid form, needs to be transported to a centralized production site, a radiopharmacy or an hospital. The transport of needed radioactive material must obey strict rules. For a radionuclide, a limit in activity that it is possible to transport has been established for each type of allowed packages. For type A package these limits are called A1 (for special form sources, i.e., certified perfectly sealed and encapsulated sources) and A2 (for non-special form sources). However, these limits can be easily reached if the activity to transport is high or if the radionuclide of interest is a “non–conventional” one. Indeed, for many radionuclides, there are no available/tabulated A1 and A2 and, in these cases, a very conservative set of values is imposed. This is in particular the case for some of the non-conventional radionuclide of interest in medicine (as for example Tb-149 or Tb-161). The non-tabulated values, and in general the A1/A2 limit, can be evaluated following the so-called Q-system and using Monte Carlo calculations. In the present work, we have used the MCNPX Monte Carlo code to evaluate dose rate values in different exposure scenarios. This has allowed us to determine A1/A2 coefficients for several non-conventional radionuclides of interest for medical applications. The developed technique can be extended easily to other radionuclides and can be adapted in case of changes in regulatory rules.

## 1. Introduction

The International Atomic Energy Agency (IAEA) Regulation for the Safe Transport of Radioactive material describes different types of packages for the transport of radioactive material in relationship to the associated risk arising from the activity and the physical form of the radioactive material contained in the package. For each radionuclide the regulation defines two values, called *A*_1_ and *A*_2_ that are used to determine the activity limit for the transportation with each type of container. In particular *A*_1_ means the activity value for special form radioactive material (indispersible solid or sealed capsule), while *A*_2_ is the activity limit for radioactive material other than special form. Type A containers allow the transport of radioisotopes with activity below *A*_1_ or *A*_2_. Type B packages are required when the activities to transport are higher than the value *A*_1_ or *A*_2_ and lower than 3,000 A_1,2_ (for shipment by plane). The definition of those activity limits for each radionuclide is made through the so-called Q-System model. It consists in a methodology in which a series of accidental exposure scenarios are used to quantify the hazard of different type of radiations. The development of the method started in the late ‘70, it has been reviewed during the years and still under study. The actual regulation, and the literature in general, still suffer of a lack of knowledge concerning those limits. For some radionuclides, indeed, there are no available/tabulated *A*_1_ and *A*_2_ and in these cases a very conservative set of values is used (Table 1). They are based on the type of the radiation emitted in the nuclide decay and on the qualitative hazard that the exposure implies; their estimation is not based on specific calculations. Moreover, in some cases they are drastically below the quantity of activity that is useful for research purposes and applications. In addition, low limits often imply the use of complex (and expensive) type of packages, like type B, whose design and homologation need competent authority approval.

**Table 1:** Activity limits for unknown radionuclides or mixture.

**Radioactive content**	***A*_1_**	***A*_2_**
	**[TBq]**	**[TBq]**
Only beta or gamma emitting nuclides	0.1	0.02
Alpha emitting nuclides (no neutrons)	0.2	9 × 10^−5^
Neutron emitting nuclides or no relevant data available	0.001	9 × 10^−5^

An impelling example of the necessity of new calculations is the case of the terbium isotopes and in particular Tb-149 and Tb-161.

Tb-149 is a low-toxicity alpha emitter with α energy of 3.97 MeV and a branching ratio of 16.7%. The remainder decay is by EC/β+ through a mean β+ energy of 0.73 MeV and a total β+ intensity of 7.1%. This isotope is used in nuclear medicine research and in particular for radioimmunotherapy studies. Since IAEA or the ADR (1) give no specific transport limit for this isotope, the generic *A*_2_ value of 9E-05 TBq (90 MBq) for alpha emitter nuclide must be used. For research purposes, involving for example the treatment of a series of mice, few hundred MBq would be needed (value of activity after chemical separation, labeling yield and decay losses) (2). The limit for the usage of a type A package is then exceeded. We will see in the next sections that the limit for the activity to transport for this isotope is not coming from the alpha but from the gamma hazards and dose rate.

Tb-161 is a low-energy beta and Auger electron emitter used for endoradiotherapeutic treatments. It has an half-life of 6.9 d and relatively low-energy β- emitted (mean energy of 0.15 MeV). Also in this case there is no tabulated values and the generic A_2_ = 0.02 TBq (20 GBq) for unknown beta emitters is applied in case of non-special form radioactive material. A single patient injection would require the use of several GBq (3). As previously said, the source must be transported from the place of production to the radiopharmaceutical lab for the chemical separation, the quality control and the labeling. Since those steps may take some days, the activity of the final product that is possible to obtain starting from maximum amount of Tb-161 to transport in a type A container won't be enough to satisfy the patients request.

It is, then, necessary to add more complete and accurate information on the transport limit of given radionulicde taking into account the real hazard coming from the nuclide spread during an accident. The non-tabulated values can be obtained combined the method defined by the Q-system with Monte Carlo simulations. The present study presents this approach by determining *A*_1_ and *A*_2_ through Monte Carlo techniques in the evaluation of the dose rate coming from the defined exposure routes, giving suggestions for possible modifications of the transportation values associated to radionuclides of potential medical interest.

## 2. Materials and Methods

### 2.1. Methodology for Calculating *A*_1_ and *A*_2_ Defined by IAEA

In the following paragraphs the main principles/hypothesis of the Q-system method are reported as described in the Appendix 1 of the IAEA Safety guide No. SSG-26 (4).

**Figure 1:**
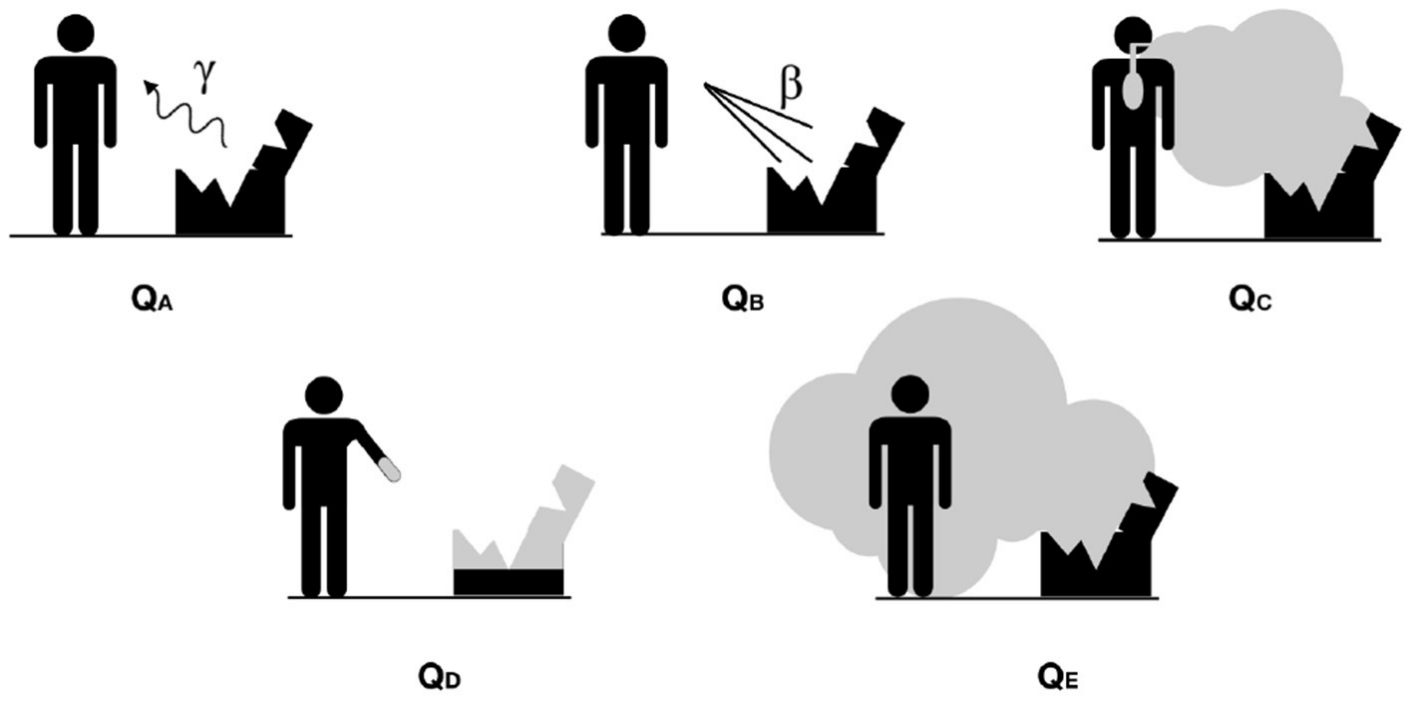
Schematic representation of the exposure pathways included in the Q system.

Under the Q-System, a series of exposure routes are considered, each of which may lead to radiation exposure (external or internal) of a person in proximity of the damaged type A package involved in a severe transport accident causing the release of some of the content. The dosimetric routes are illustrated in the Figure 1 and led to five limit values, called, indeed, “Q values”:

- *Q*_*A*_ for external dose due to photons,
- *Q*_*B*_ for external dose due to beta emission,
- *Q*_*C*_ for internal dose due to inhalation,
- *Q*_*D*_ for skin contamination and ingestion dose from beta emission,
- *Q*_*E*_ for submersion dose due to gaseous isotopes.

Special form radioactive materials are able to retain eventual gas or fragments of the source following an accident due to their characteristics of certified sealed capsule. For this reason, the scenarios defined by *Q*_*C*_, *Q*_*D*_ and *Q*_*E*_ values are not relevant. Consequently, the *A*_1_ value, for special form materials, corresponds to the minimum value between *Q*_*A*_ and *Q*_*B*_.

For non-special form radioactive materials, instead, the source is not necessarily sealed: *A*_2_ is the minimum among the five Q values, since all the scenarios are possible.

#### 2.1.1. Calculation of *Q*_*A*_: External Dose Due to Photons

The *Q*_*A*_ value is determined by the consideration of the external radiation dose due to the gamma or the X-rays to the whole body of a person exposed near a type A package following an accident. In this scenario the source is considered placed at 1 m from the person and the shield is assumed completely lost during the accident. In the revised Q-system, the information from the gamma emission spectrum for the radionuclides are coming from the ICRP Publication 38 (1984) and for the calculations the source is considered isotropic and pointlike. The *Q*_*A*_ values are given by:


(1) $$\begin{array}{l} Q_{A}=\frac{D/t}{DRC_{\gamma }A}C \end{array}$$

where D is the reference dose of 0.05 Sv (50 mSv), t is the exposure time of 0,5 h (30 min), DRC_γ_ is the effective dose rate coefficient for the radionuclide, C is the conversion factor determining the units for *Q*_*A*_ (10^−12^ since Q are given in TBq) and A is the activity of the source (1 Bq).

Including all these values in the previous equation we obtain:


(2) $$\begin{array}{l} Q_{A}\left(TBq\right)=\frac{10^{-13}}{{ė}_{pt}} \end{array}$$

where: ė_*pt*_ is the effective dose rate coefficient for the radionuclide at a distance of 1 m in air (Sv Bq^−1^ h^−1^). A (non-exhaustive) list of dose and dose rate coefficients may be found in Table II.2 Appendix II of the IAEA Safety Guide (4).

The dose rate coefficient has been calculated from the following equation:


(3) $$\begin{array}{l} {ė}_{p}=\frac{C}{4\pi d^{2}}\underset{i}{\sum }{\left(\frac{e}{X}\right)}_{E_{i}}Y_{i}E_{i}{\left(\frac{\mu_{en}}{\rho }\right)}_{E_{i}}e^{-\mu_{i}d}B\left(E_{i},d\right) \end{array}$$

where:

– (e/X)_*Ei*_ is the relationship between the effective dose and exposure in free air (Sv R^−1^; R stands for Rontgen unit measure of the exposure, 1R = 2.58 x 10^−4^C kg^−1^);
– Y_*i*_ is the yield of photons of energy E_*i*_ per disintegration of the radionuclide (Bq s)^−1^;
– E_*i*_ is the energy of the photon (MeV);
– d is the distance in air (1 m) from the source;
– (_μ_*en*_/ρ)*E*_*i*__ is the mass energy absorption coefficient in air for photons of energy *E*_*i*_ (cm^2^ g^−1^);
– μ_*i*_ is the linear attenuation coefficient in air for a photon of energy E_*i*_ (cm^−1^);
– B(E_*i*_,d) is the air Kerma buildup factor for photons of energy E_*i*_ and distance d of 1m;
– C is a constant given by the above units.

The values of (e/X)_*E*_*i*__ are obtained by interpolating the data from ICRP Publication 51 (5) for photons in the range 5 keV to 10 MeV.

#### 2.1.2. Calculation of *Q*_*B*_: External Dose Due to Beta Emitters

The *Q*_*B*_ value is determined as the beta dose to the skin of a person exposed following an accident involving a type A package. The shielding of the transport package is not assumed to be completely lost in the accident as for the previous case, but a residual shielding factor for beta emitters (such as the source protection elements, package debris, etc.), included in the 1985 Edition of the Transport Regulations, is considered. Contrary to the gamma radiation, the electrons of the source can strongly interact with the materials around it and so the presence of a residual shielding can contribute to absorb the radiation (and to reduce part of the dose).

In the revised Q system, *Q*_*B*_ is calculated by using the complete beta spectra for the radionuclides of ICRP Publication 38 (6). The spectral data for the nuclide of interest are used to evaluate skin dose rate per unit activity of a monoenergetic electron emitter.

Cross et al. (7, 8) *Q*_*B*_ is given by:


(4) $$\begin{array}{l} Q_{B}=\frac{D/t}{DRC_{\beta }}C \end{array}$$

where:

– D is the reference dose to a particular organ (here the skin) of 0.5 Sv;
– t is the exposure time of 0.5 h;
– DRC_β_ is the equivalent skin dose rate coefficient for the radionuclide;
– C is a conversion factor that determines the units for *Q*_*B*_ (10^−12^ since the Q are given in TBq).

Thus, including in the equation the correct factors, the *Q*_*B*_ can be calculated from:


(5) $$\begin{array}{l} Q_{B}\left(TBq\right)=\frac{1\times 10^{-12}}{{ė}_{\beta }} \end{array}$$

where ė_β_ is the equivalent skin dose rate coefficient for beta emission at a distance of 1 m in air from the self-shielded material (Sv Bq^−1^ h^−^1). Dose and dose rate coefficients may be found in Table II.2 of Appendix II (4).

The dose rate coefficient is defined as:


(6) $$\begin{array}{l} {ė}_{\beta }=\frac{1}{SF_{\beta_{max}}}J_{air}C \end{array}$$

with:

– SF_β_*max*__ is the shielding factor computed at the maximum energy of the beta spectrum (see more details below);
– J_*air*_ is the dose at 1 m (in air) per disintegration (MeV g^−1^ Bq^−1^s^−1^);
– C is a numerical conversion constant.

The factor J_*air*_ is computed as:


(7) $$\begin{array}{l} J_{air}=\frac{n}{4\pi \rho r^{2}}\int_{0}^{E_{max}}N\left(E\right)j\left(r/r_{E},E\right)\left(E/r_{E}\right)dE \end{array}$$

where:

– n is the number of beta particles emitted per disintegration;
– N(E) is the number of electrons emitted with energy between E and E+dE (Bq^−1^s^−1^);
– j(r/r_*E*_, E) is the dimensionless dose distribution that represents the fraction of emitted energy deposited in a spherical shell of radius r/r_*E*_;
– r/r_*E*_ + d(r/r_*E*_) is as tabulated by Cross et al. (7, 8).

Finally, a comment should be made about the treatment of positron annihilation radiation and conversion electrons in the determination of Q values. The latter are treated as monoenergetic beta particles, and weighted according to their yields. In the case of annihilation radiation this has not been included in the evaluation of the beta dose to the skin since it contributes only to an additional few per cent to the local dose to the skin basal layer. However, the 0.511 MeV gamma rays are included in the photon energy per disintegration used in the derivation of *Q*_*A*_.

#### 2.1.3. Considerations on the Shielding Factor (SF) Calculation

The self- shielding of the package was taken to be a smooth function of the maximum energy of the beta spectrum (*E*_β, *max*_):


(8) $$\begin{array}{l} SF=e^{\mu d} \end{array}$$

Where d is the thickness of the absorber equal to 150 mg/*cm*^2^ and μ [*cm*^2^/mg] is the apparent absorption coefficient given by the following empirical equation:


(9) $$\begin{array}{l} \mu =0.017{\left(E_{\beta ,max}\right)}^{-1.14} \end{array}$$

The method assumes a very conservative shielding factor of 3 for beta emitters of maximum energy ≥2 MeV, and based on an absorber of approximately 150 mg cm^−2^ thickness.

#### 2.1.4. Calculation of *Q*_*C*_ : Internal Dose via Inhalation

The *Q*_*C*_ value is connected to the inhalation risk, supposed to be negligible for special form radioactive materials. Following an accident, a portion of the material escapes from the package becoming airborne and leading to a dose for the worker via inhalation. This scenario includes accidents occurring both indoors and outdoors. Potentially the most severe type of accident for many type A packages is the combination of mechanical damage with a fire, producing relatively large sized particles that may be inhaled.

Data on the respirable aerosol fractions produced under accidental conditions are generally sparse and are only available for a limited range of materials.

In the Regulation [Appendix 1 of International Atomic Energy Agency (IAEA)] (4), it is assumed that 10^−6^ of the package contents has escaped as a result of an accident and that this quantity of material is inhaled by a person on the scene. It represents a combination of releases typically in the range up to 10^−3^-10^−2^ of the package contents as a respirable aerosol, combined with an uptake factor of up to 10^−4^-10^−3^ of the released material.

Considering also the limiting doses, this leads to an expression for the contents limit based on inhalation of the form:


(10) $$\begin{array}{l} Q_{C}=\frac{D}{1\times 10^{-6}DC_{inh}}C \end{array}$$

where:

– D is the reference dose of 0.05 Sv;
– 10^−6^ is the fraction of the inhaled content of the package;
– DC_*inh*_ is the dose coefficient for inhalation;
– C is the conversion factor that determines the units of *Q*_*C*_ (10^−12^ TBq/ Bq).

Using these factors and coefficients, the *Q*_*C*_ value can be calculated as follow:


(11) $$\begin{array}{l} Q_{C}\left(TBq\right)=\frac{5\times 10^{-8}}{{ė}_{inh}} \end{array}$$

where ė_*inh*_ is the effective dose coefficient for inhalation of the radionuclide (Sv/Bq). Values for ė_*inh*_ may be found in Tables II, III of Appendix II the Safety Series n.115 (9), while dose and dose rate coefficients may be found in Table II.2 of Appendix II (4).

#### 2.1.5. Calculation of *Q*_*D*_: Skin Contamination and Ingestion Dose

The *Q*_*D*_ value for beta emitters is determined by the beta dose to the skin of a person contaminated with radioactive material as a consequence of handling a damaged type A package. The model proposed within the Q system assumes that 1% of the package contents are spread uniformly over an area of 1 m^2^; handling of the debris is assumed to result in contamination of the hands to 10% of this level (10). It is further assumed that the exposed person is not wearing gloves but would recognize the possibility of contamination or wash the hands within a period of 5 h.

The dose rate limit for the skin is fixed to 0.1 Sv/h based on a 5 h exposure period.

The values for *Q*_*D*_ have been calculated using the continuum beta spectra and discrete electron emissions for the radionuclides as tabulated by the ICRP 38 and 51 (5, 6).

*Q*_*D*_ is given by:


(12) $$\begin{array}{l} Q_{D}=\frac{D}{10^{-3}\times DRC_{skin}\times t}C \end{array}$$

where:

– D is the reference dose to a particular organ (skin in this case) of 0.5 Sv;
– 10^−3^ is the fraction of the package content distributed per unit area of the skin (m^−2^);
– DRC_*skin*_ is the equivalent skin dose rate coefficient for skin contamination;
– t is the exposure time of 1.8 × 10^4^ s (5 h);
– C is a conversion factor that determines the units for *Q*_*D*_ (set to 1).

With those factors, *Q*_*D*_ can be evaluated as:


(13) $$\begin{array}{l} Q_{D}\left(TBq\right)=\frac{2.8\times 10^{-2}}{{ḣ}_{skin}} \end{array}$$

where ḣ_*skin*_ is the equivalent skin dose rate per unit activity and unit area of the skin (Sv s^−1^ TBq^−1^ m^2^). dose and dose rate coefficients may be found in Table II.2 of Appendix II (4).

The models used in deriving the *Q*_*D*_ values here may also be employed to estimate the possible uptake of radioactive material via ingestion, but since the dose per unit intake via inhalation is generally of the same order as, or greater than, the one via ingestion (11), the inhalation pathway will normally be limiting for internal contamination under the Q system.

#### 2.1.6. Calculation of *Q*_*E*_: External Exposure in Air

For gaseous isotopes which do not become incorporated into the body, such as noble gases, an additional Q value, *Q*_*E*_, is determined from the dose from external irradiation in a cloud of gas.

Both the effective dose and skin dose must be calculated in this case, assuming that:

1. the entire package contents is released;
2. the release occurred in a room or cargo handling bay of 300 *m*^3^ of volume, area in wich the person is exposed;
3. there are 4 air changes per hour within the room.

These assumptions led to an initial airborne concentration of *Q*_*E*_/300 Bq *m*^−3^, which decreased exponentially at a rate of 4 *h*^−1^. The average activity concentration in air over the exposure time (0.5 h) was 1.44 10^−3^ *Q*_*E*_ (*m*^−3^). Submersion dose coefficients for effective and skin dose are given in the Federal Guide n.12 (12) and are listed in IAEA TS-G-1.1 (4).

*Q*_*E*_ values for effective dose is calculated as follows:


(14) $$\begin{array}{l} Q_{E}=\frac{DL_{eff}}{TIAC\;h_{subeff}}C \end{array}$$

While the *Q*_*E*_ values for the dose to the skin (TBq) is calculated as:


(15) $$\begin{array}{l} Q_{E}\left(TBq\right)=\frac{DL_{skin}}{TIAC\;h_{subskin}}C \end{array}$$

where:

– *DL*_*eff*_ and *DL*_*skin*_ are the dose criteria for effective dose (0,05 Sv) and equivalent dose to the skin (0,5 Sv), respectively;
– TIAC is the time-integrated activity concentration in air per unit activity released which was set to 2.6 Bq s *m*^−3^ per Bq;
– C is the conversion factor that determines the units for *Q*_*E*_ (10^−12^);
– *h*_*subeff*_ and *h*_*subskin*_ are the submersion dose coefficient for effective dose and skin equivalent dose, respectively (Sv Bq^−1^s^−1^m^3^), provided by IAEA TS-G-1.1 (4).

The *Q*_*E*_ value is the lower of two values calculated for the effective and skin equivalent dose.

#### 2.1.7. Special Considerations

- ***Treatment of the progeny:***
- The Q system assumed a maximum transport time of 50 days, and thus radioactive decay products with half-lives lower than 10 days were assumed to be in secular equilibrium with their longer lived parents. In such cases, the Q values were calculated for the parent and its progeny, and the limiting value was used in determining *A*_1_ and *A*_2_ of the parent. In cases where a daughter radionuclide has a half-life either greater than 10 days or greater than the one of the parent nuclide, such progeny, with the parent, are considered to be a *mixture*. The *A*_1_ and *A*_2_ values for mixtures of n radionuclides are determined as follow (4):
  (16) $$\begin{array}{l} X_{m}=\frac{1}{\sum_{i}^{n}\frac{f\left(i\right)}{X\left(i\right)}} \end{array}$$
- where:
  – *X*_*m*_ is the derived value of *A*_1_ or *A*_2_ in case of a mixture;
  – f(i) is the fraction of activity or activity concentration of the radionuclide i in the mixture;
  – X(i) is the *A*_1_ or *A*_2_ value for the radionuclide i.
- ***Rounding method:***
- The Q values are quoted to 2 significant digits whereas *A*_1_ and *A*_2_ values are rounded up or down to the nearest significant figure.

## 3. Calculation of *A*_1_ and *A*_2_ with Monte Carlo method

The methodology described in the previous sections implies the use of analytic formulae or empiric coefficients and relies in some cases on the approximation of integral equations. Moreover, the information on the isotopes' spectra are based on old libraries dated 1984-94.

A good alternative is represented by the use of Monte Carlo simulations to evaluate directly the dose rate parameters to use in the formulae for the calculation of the Q values: ė_*pt*_, ė_*b*_, ḣ_*skin*_, ė_*inh*_.

This method avoids the solution of complex equations and takes into account all the phenomena involved in the interaction of the source's particles with the matter and the surrounding air, giving a realistic evaluation of the dose in the single accidental scenarios. It will include the recent nuclear physics interaction cross sections of the particles as well as effects like Bremsstrahlung that has not been fully included in the current Q-system. However, the basic principles, like the geometrical factors and the radiological criteria of the current Q system, remain.

The Monte Carlo computer program MCNPX (13) has been used for these calculations. The information relative to the decay spectra of the single isotopes are coming from the ICRP 107 publication (14).

Each nuclide is characterized by a typical spectrum of emission. A procedure that allows a fast calculation for each nuclide without the need to set a different MC code for each of them has been used: the dose rate values is computed for monoenergetic particles sources; then, using the typical spectra characteristics (energy distribution and branching ratio of the particles emitted in the decay), the effective dose rate is associated to each radionuclide.

The applied method is similar in all the cases/scenarios and it is composed by the following main steps:

**Step 1:**

–      The geometry reproducing as close as possible the accidental scenario described by the Regulation for the single Q value is modeled in the MCNPX code;

**Step 2:**

–      A pointlike source of beta or gamma particles of 1 Bq is set up (in the origin) and its emission considered isotropic and monoenergetic. The spectra of energies simulated goes from 0.01 to 5 MeV for gamma and 0.1 to 5 MeV for electrons and positrons. The number of primaries used is 1.0E+07.
–      The dose rate for the defined active/detection area and associated to the single energy with emission probability of 100% is evaluated using the MCNPX F8 tally;

**Step 3:**

–      Using the spectra of each isotope, the dose rates associated to the single energies are calculated;
–      The dose rate for each i-th particle energy composing the spectra is weighted by the relative effective Branching Ratio;
–      When the emitted particle energy is not present in the simulated data set, a linear interpolation is done for that particular energy bin;
–      For monoenergetic spectra the total dose rate is given by the arithmetic sum of the single dose rates weighted by the probability of decay:
–      In case of continuum spectra (i.e., beta emission) the dose rate is coming from the trapezoidal integration rule of the data set.
–      If the isotope is characterized by both monoenergetic and a continuous spectra, the dose rate is the sum of the two components.

**Step 4:**

–      The obtained dose rate coefficient is used to calculate the Q value under study using the formulae presented in the previous section.

In all calculations the dose rate is relative not only to the primary particles emitted from the source, but also to the effect of the secondary particles, coming from the elastic and inelastic scattering with the surrounding materials. Unlike the analytical calculations, these effects can be easily taken into account using the Monte Carlo method.

- The evaluation of h_*skin*_, involved in the calculation of the *Q*_*C*_ value, is linked to the dose rate released to the organs of the respiratory tract. The complexity related to the needs to understand the fractional deposition and the chemical affinity in each sector of the respiratory organs for each radionuclide, led us to use the values of h_*skin*_ defined in the ICRP119 publication (15) for the calculations.
- The recalculation of the dose coefficient *h*_*subeff*_ and *h*_*subskin*_, involved in the calculation of *Q*_*E*_ value, is not of interest (is not an objective) for this study. The main reason is that the gaseous form of radioactive medical isotopes to transport is very rare. Moreover, the *Q*_*E*_ calculations imply the knowledge of the isotopes concentration on the air volume of the room with the time and the need to simulate the dose for the general human phantom.

This study focuses in particular on the re-calculation of the *Q*_*A*_, *Q*_*B*_ and *Q*_*D*_ values, keeping the ones defined in the Regulation for *Q*_*C*_ and *Q*_*E*_ unchanged for the final comparison.

The Monte Carlo method have been initially tested for a *control group* of Isotopes for whom the dose coefficients that appear in the equations for the Q values are tabulated in the IAEA Safety Guide. A comparison between the listed coefficients and the ones simulated in this study have been done to validate the method.

The procedure have been then applied to evaluate the dose rate coefficients (ė_*pt*_, ė_*b*_, ḣ_*skin*_, ė_*inh*_) for some nuclides who present non-tabulated Q values and generic limits of transport.

In the following sections all the parameters and the modeling approach used in the Monte Carlo simulations for each accidental scenario defined by the Q-system will be described.

### 3.1. Calculation of *Q*_*A*_ With the MC

As defined by the IAEA method, the ė_*pt*_ dose rate is given by the whole body exposure to gamma or the X-Rays of a person as consequence of an accident.

**Figure 2:**
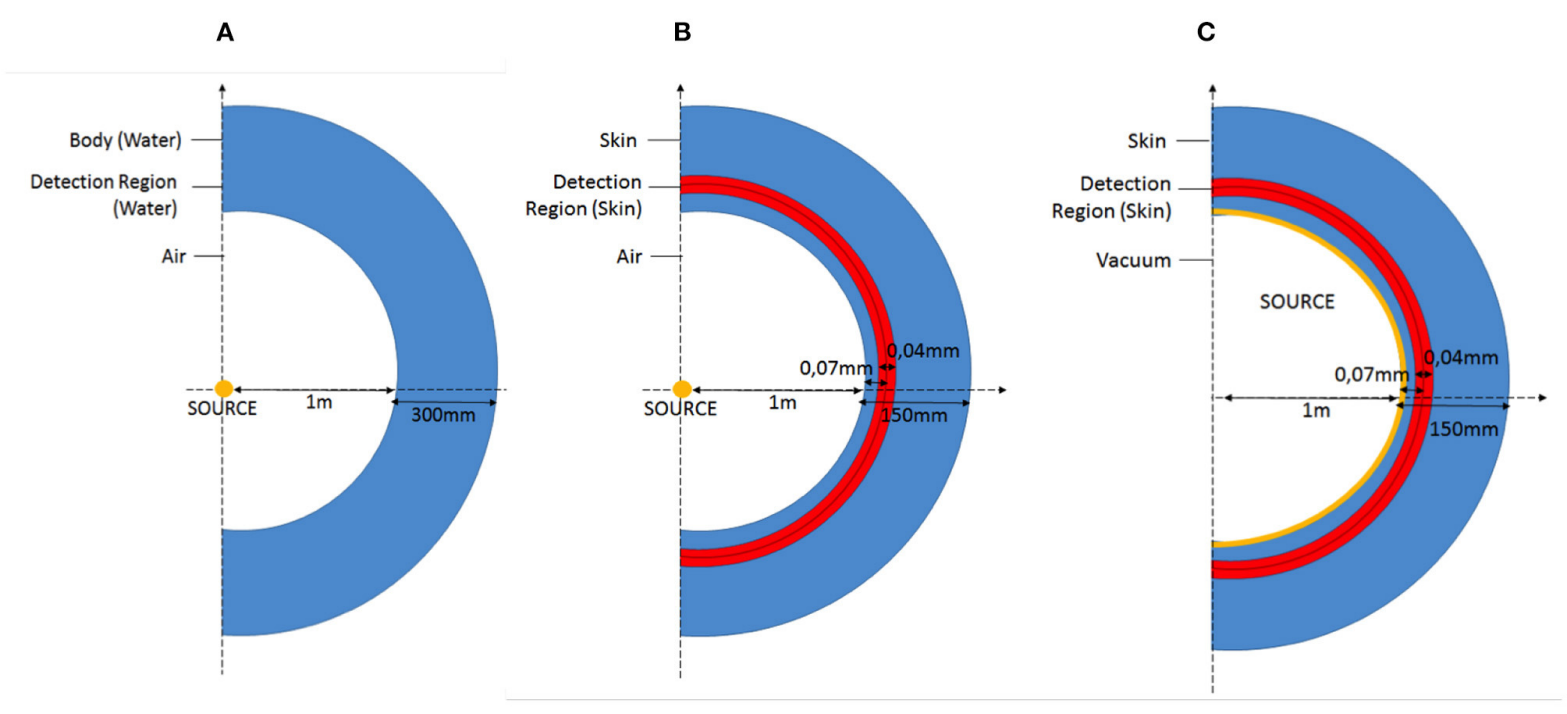
Scheme of the geometry reproduced with MNCPX representing the accidental scenario involved in the *Q*_*A*_ **(A)**, *Q*_*B*_ **(B)**, and *Q*_*D*_ **(C)** simulations.

The scenario described by the IAEA method and modeled with MCNPX is reported in the Figure 2A.

The gamma source, isotropic and monoenergetic, is placed in the center of the axis. The person (representing our active area/detector) is placed, in air, at 1 m from the source: the active area is represented by a spheric shell with inner radius of 1 m and thickness of 0,30 m composed by water. The reason of this material choice is due to the similar density and composition of water with the human body (Table 2). The thickness of 30 cm has been chosen as mean thickness of the human body. The cylindrical symmetry of the simulated geometry is made to increase the number of particles reaching the detection area and reduce consequentially the variance of the results.

**Figure 3:**
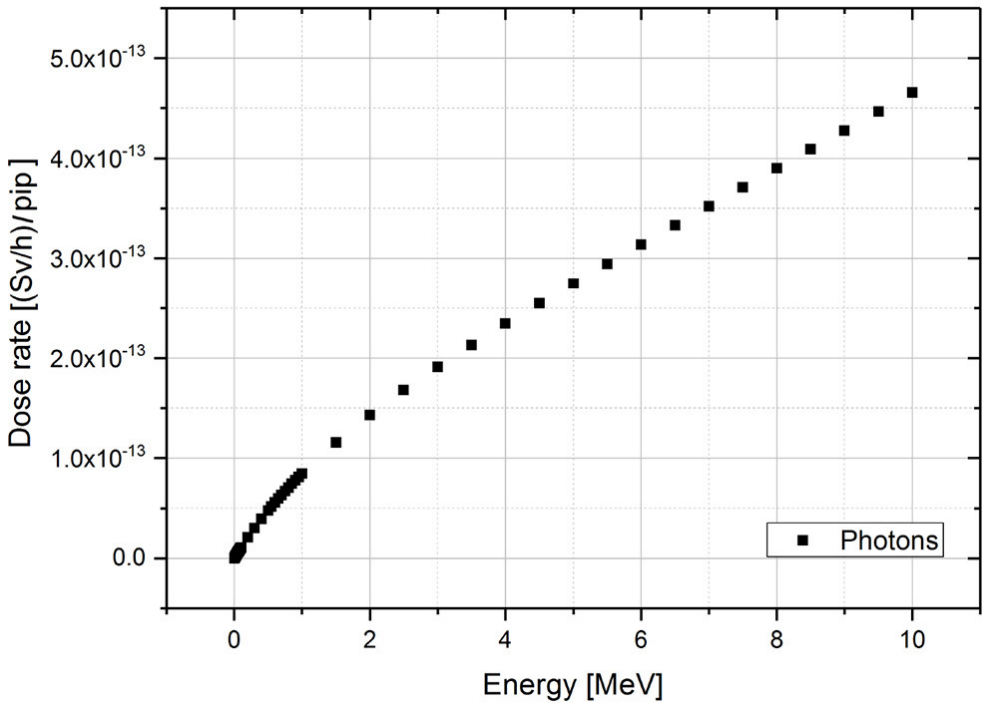
Dose rate results of MCNPX simulations for monoenergetic gamma sources per incident particle (pip). The range of simulated energies goes from 0.01 to 10 MeV.

The values of the simulated dose rates with the energy for the monoenergetic gamma sources are plotted in the Figure 3.

**Table 2:** Composition of materials used for the gamma dose simulations in MCNPX.

**Material**	**Weight fraction**	**Density**
	**[%]**	**[g cm^**−3**^]**
Air	Ar: 1.28	0.001205
	O: 23.18	
	C: 0.012	
	N: 75.53	
Water	H: 11.2	1
	O: 88.8	

Considering the gamma spectra for each isotope (energy and associated branching ratio), the ė_*pt*_ dose rate factor is given by the sum of the dose rate associated to the single energies (Ḋ(*Ei*)) weighted by their relative probability of emission (I(Ei)).


(17) $$\begin{array}{l} {ė}_{pt}=\overset{n}{\underset{i=1}{\sum }}I\left(E_{i}\right)Ḋ\left(E_{i}\right) \end{array}$$

Using the Equation 2 the *Q*_*A*_ factor is then evaluated. The results of simulation for the dose rate coefficient ė_*pt*_ and the relative *Q*_*A*_ values for the chosen control group and for the other nuclides of interest are reported in the Table 3.

**Table 3:** Results of the Q values obtained using the simulated dose rate coefficients and the ones listed in the IAEA Safety guide (4).

	**Radionuclide**	***Q***_*****A*****_		***Q***_*****B*****_		***Q***_*****C*****_		***Q***_*****D*****_	
				**TBq**					
		**MCNPX**	**IAEA**	**MCNPX**	**IAEA**	**MCNPX**	**IAEA**	**MCNPX**	**IAEA**
Control group	Be-7	2.09E+01	2.10E+01	1.82E+06	1.00E+03	9.62E+02		2.90E+00	1.00E+03
	Na-22	5.29E-01	5.00E-01	2.71E+00	3.80E+00	2.50E+01	3.85E+01	6.96E-01	6.50E-01
	Na-24	3.48E-01	3.00E-01	2.47E-01	2.00E-01	1.72E+02	1.70E+02	7.02E-01	6.00E-01
	Ca-47	1.04E+00	2.70E+00	5.16E-01	3.70E+01	1.77E+01	2.00E+01	3.54E-01	3.30E-01
	Co-58	9.89E-01	1.10E+00	8.95E+01	7.80E+02	3.57E+01	2.50E+01	4.01E+00	3.80E+00
	Co-60	5.95E-01	4.50E-01	3.01E+02	7.30E+02	2.07E+00	1.70E+00	9.23E-01	9.70E-0
	Sr-82	9.53E-01	9.70E-01	2.82E-01	2.40E-01	5.00E+00		4.02E-01	5.90E-01
	Y-90	8.21E+05	1.00E+03	2.68E-01	3.20E-01	3.30E+01		7.43E-01	5.90E-01
	Cs-137	1.57E+00	1.80E+00	2.49E+00	8.20E+00	7.46E+00	1.00E+01	6.66E-01	6.30E-01
	At-211	2.15E+01	2.50E+01	1.56E+02	1.00E+03	4.55E-01	5.10E-01	2.33E+02	4.40E+02
Other radionuclides	Cu-61	1.12E+00	-	1.09E+00	-	4.17E+02	-	1.12E+00	-
	As-71	1.82E+00	-	1.09E+01	-	1.00E+02	-	1.67E+00	-
	Se-72	6.09E-01	-	1.39E-01	-	5.43E+01	5.10E-01	4.17E-01	-
	Nd-140	3.16E+01	-	4.75E-01	-	-	-	1.46E+00	-
	Tb-152	7.94E-01	-	7.53E-01	-	-	-	2.81E+00	-
	Tb-155	5.27E+00	-	1.24E+03	-	2.00E+02	-	3.81E+00	-
	Tb-156	5.99E-01	-	3.24E+01	-	3.57E+01	-	1.23E+00	-
	Tb161	7.11E+00	-	1.86E+02	-	4.17E+01	-	7.58E-01	-
	Tm-166	6.23E-01	-	1.13E+01	-	1.79E+02	-	1.70E+00	-
	Yb-166	5.88E-01	-	1.02E+01	-	4.20E+01	-	1.43E+00	-
	Tb-149	8.56E-01	-	2.40E+00	-	1.61E+01	-	2.31E+00	-
	Bi-213	5.29E-01	-	4.54E-01	-	1.22E+00	-	6.15E-01	-

### 3.2. Calculation of *Q*_*B*_ With the MC

The *Q*_*B*_ value is determined by the beta dose to the skin of a person exposed during an accident involving a type A package containing special form material. A residual shielding factor (SF) for beta emitters is considered.

The geometry reproduced in MCNPX is reported in Figure 2B. The person exposed is at 1 m from the source. In this case the dose to the skin is of interest, so the active area is a spheric shell with thickness of 0.04 mm and depth of 0.07 mm. It corresponds to the position of the layer of the skin called dermis, containing blood vessels and lymph nodes.

The composition of the skin used for the calculation is the one reported in the International Commission on Radiation Unit and measurements (ICRU) (16), while air composition is the same used in the *Q*_*A*_ calculation (Table 2).

**Figure 4:**
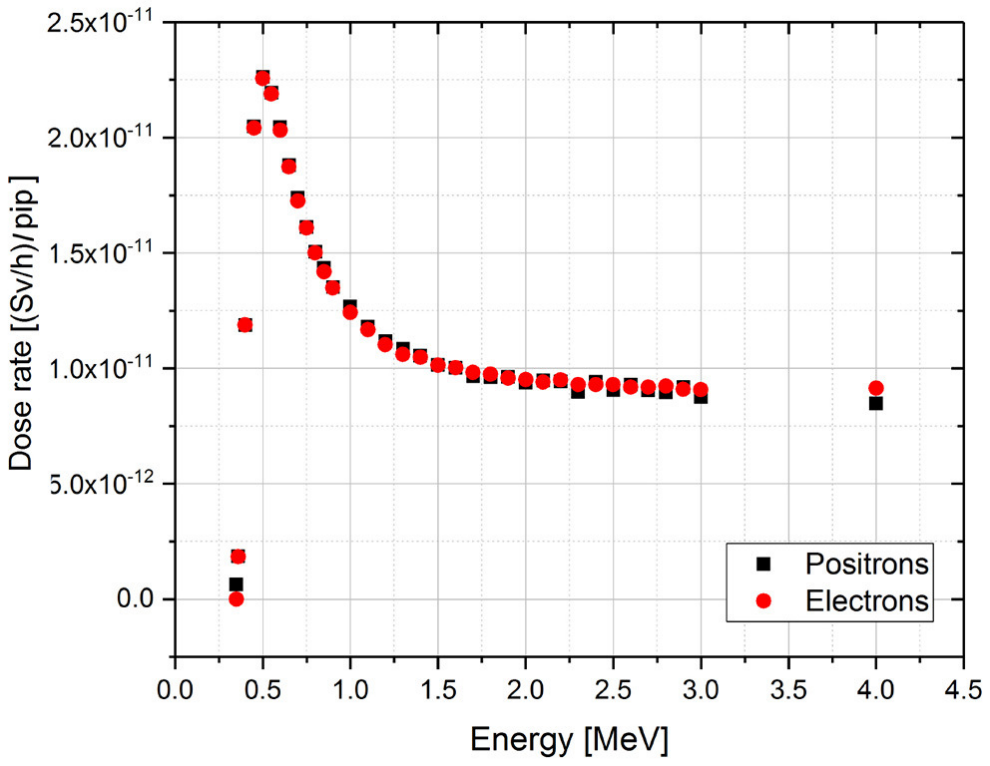
Dose rate results of the MCNPX simulations for monoenergetic electron and positron sources per incident particle (pip). The simulated energy range goes from 0.36 to 4 MeV.

The simulated dose rate to the skin for the single energy positron and electron source is reported in the graph below (Figure 4). The energy of 0,36 MeV has been chosen as lower energy limit. It corresponds to the minimum energy for a e- particle to have a range comparable with the source-water layer distance, i.e., 1 m in this case.

For energy values 0.3–0.5 MeV we can observe that the dose rate increases up to a peak. Here the source-detector distance (1 m in air + 0.07 mm of water + 0.04 mm of water detector in this case) corresponds to the maximum depth at which the incident electrons with those energies are repeatedly scattered and penetrate into the target while losing their energy. Increasing the energy, the electrons ranges become higher and they will go through the detector depositing only a fraction of their energy. Above 2 MeV the behavior can be assumed linearly decreasing. The choice of the binning reflects this behavior: small bin is used to sample the peak region and a larger one in the linear decreasing region and at the end of the curve tail.

Positrons and electrons have basically the same behavior (same deposited energy) in the skin tissue. There is a density effect correction coefficient that differentiates the collision stopping power of the two charged particles (17). For positrons, annihilation occurs leading to the production of two 511 keV gammas which have been already taken into account in the gamma spectrum characterizing the *Q*_*A*_ value.

The dose rate is given by the result of the sum of two factors: the dose coming from the continuum beta specrum (${{ė}_{b}}^{cont}$) and the dose given by the monoenergetic electrons emitted during the decay (${{ė}_{b}}^{mono}$). A coefficient dependent to the maximum beta energy, linked to the residual shielding (SF) and defined as in the paragraph 2.1.3 is also included:


(18) $$\begin{array}{l} {ė}_{b}=SF\left({{ė}_{b}}^{cont}+{{ė}_{b}}^{mono}\right) \end{array}$$

For the evaluation of the first factor $e_{b}^{cont}$, the single dose rate values are weighted by their branching ratio and integrated using the trapezoidale rule:


(19) $$\begin{array}{l} {ė}_{b}^{cont}=\sum_{i=1}^{n}\frac{\left(BR_{n}{Ḋ}_{n}+BR_{n-1}{Ḋ}_{n-1}\right)}{2}\Delta E_{i} \end{array}$$

The second factor $e_{b}^{mono}$ is given by the sum of the dose rate of the single energies weighted by their branching ratio:


(20) $$\begin{array}{l} e_{b}^{mono}=\overset{n}{\underset{i=1}{\sum }}BR_{i}Ḋ\left(E_{i}\right) \end{array}$$

In both Equations (19 and 20) the dose rate values are weighted by the probability of emission (BR).

The calculation of the adimensional SF follows the method established in the IAEA regulation: if the isotope under study presents particles with energies higher than 2 MeV, the shielding factor is set to 3, otherwise it will depend on the maximum beta energy of the beta spectra (Equation 8). In case the isotope presents only monoenergetic electrons, a shielding factor of 3 is chosen a priori, independently from the spectra.

### 3.3. Calculation of *Q*_*D*_ With the MC

The *Q*_*D*_ factor is related to the accidental scenario in which the dose is transferred to the person due to the handling of the damaged Type A package.

The geometry reproduced in the code is reported in the Figure 2C.

The source is now at contact with the skin and the area of detection is still represented by a spherical shell with thickness of 0.04 mm and at a depth 0.07 mm. The skin composition is the same than the one reported in the Table 2.

The method of the h_*skin*_ dose factor calculation is similar to the one used for the coefficient ė_*b*_ except for the absence of the shielding factor effect.

**Figure 5:**
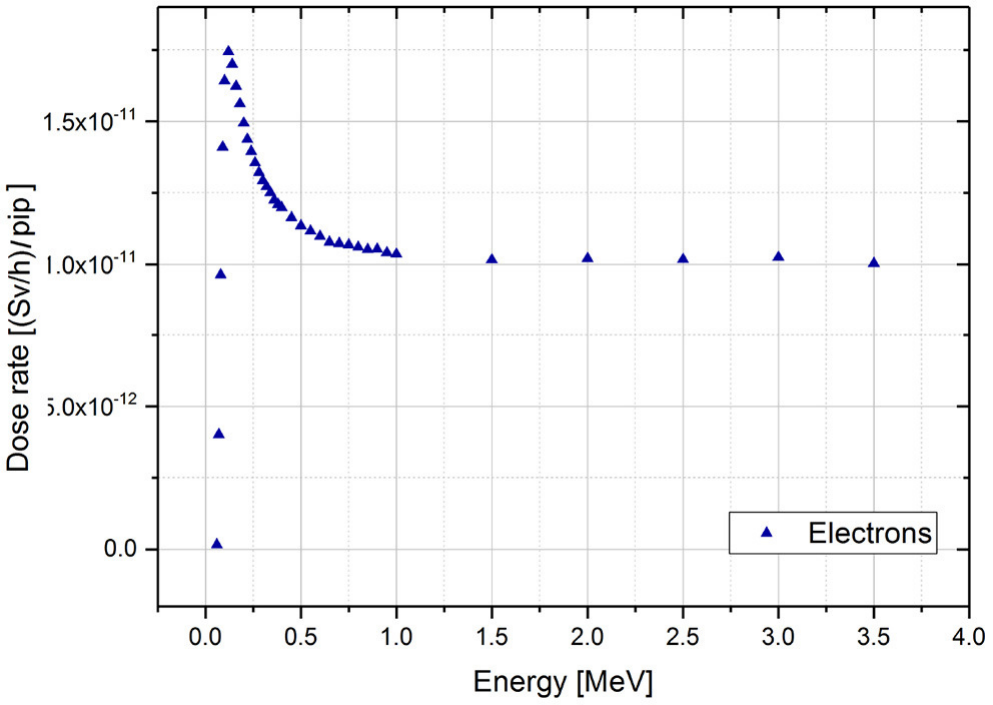
Dose rate results of the MCNPX simulations for monoenergetic electron sources per incident particle (pip). The results for the e+ source provide a dataset that differentiates from the one of the e- of a factor minor that the 1% and it has not been reported in the graph for simplicity.

As first step, the dose rate for the single energies (with 100% of branching ratio) is evaluated. The results of the simulations are reported in Figure 5.

The range of chosen energies goes from 0.06 (minimum energy to have electrons with range comparable to the skin thickness) to 4 MeV.

As for the previous ė_*b*_ case, it is possible to distinguish three regions in the dose rate behavior as a function of the energy.

In the first region, the dose rate increase up to a maximum value corresponding to the energy for which the electrons range is equal to the source-skin derma distance. For higher energies, the range of the electrons increases at the expense of the deposited dose in the detection area. Then the second region is characterized by an exponential decay of the dose rate values. Starting from 1 MeV, it is possible to assume a linear decreasing behavior, corresponding to the third region. The choice of the energy bin for the spectra reflects this trend: small bins are used to sample the first two regions, while a larger one is used for the curve tail.

Subsequently, the spectra of the isotopes under study are retrieved. Once again, the dose rate coming from the (n) monoenergetic electron of the spectra is given by the sum of the single contribution to the dose (Ḋ(Ei)) weighted by the relative probability of emission (BR). The contribution to the dose coming from the continuum spectra is given by the trapezoidal integration of the single contribution always weighted by their relative probability of emission.


(21) $$\begin{array}{l} h_{skin}=h_{skin}^{cont}+h_{skin}^{mono} \end{array}$$

where:


(22) $$\begin{array}{l} h_{skin}^{cont}=\sum_{i=1}^{n}\frac{\left(BR_{n}{Ḋ}_{n}+BR_{n-1}{Ḋ}_{n-1}\right)}{2}\Delta E_{i}\;\text{and}\\ h_{skin}^{mono}=\overset{n}{\underset{i=1}{\sum }}BR_{i}Ḋ\left(E_{i}\right) \end{array}$$

### 3.4. Results of the *A*_1_ and *A*_2_ Limits With the Monte Carlo Technique

The entire set of results of the Monte Carlo method described in the previous paragraph are summarized in the Tables 3–5, reporting, respectively the dose rate coefficients ė_*p*_, ė_*b*_, *h*_*skin*_, ė_*d*_, the relative Q values and the *A*_1_ and *A*_2_ limits compared with the ones specified in the IAEA Safety Guide. Three graphs can be useful to visually compare the Monte Carlo sets of data with the Regulatory ones and make some conclusions.

**Table 4:** Results of the dose coefficients obtained with the Monte Carlo method.

	**Radionuclide**	**Daughter**	**Decay mode**	ė_*****pt*****_		***e***_*****b*****_		***e***_*****inh*****_		***h***_*****skin*****_	
				**Sv** ***Bq***^**−1**^***h***^**−1**^		**Sv** ***Bq***^**−1**^***h***^**−1**^		**Sv** ***Bq***^**−1**^		**Sv** ***m***^**−2**^***TBq***^**−1**^***s***^**−1**^	
				**MCNPX**	**IAEA**	**MCNPX**	**IAEA**	**MCNPX**	**IAEA**	**MCNPX**	**IAEA**
Control group	Be-7		EC	4.78E-15	4.80E-15	5.48E-19	1.00E-15	5.20E-11		9.64E-03	2.80E-05
	Na-22		EC B+	1.89E-13	2.00E-13	3.69E-13	2.60E-13	2.00E-09		4.02E-02	4.20E-02
	Na-24		B-	2.87E-13	3.30E-13	4.05E-12	5.00E-12	2.90E-10		3.99E-02	4.70E-02
	Ca-47	Sc-47	B-	9.61E-14	3.70E-14	1.94E-12	2.70E-14	2.83E-09		7.92E-02	8.40E-02
	Co-58		EC B+	1.01E-13	9.10E-14	1.12E-14	1.30E-15	2.00E-09		6.97E-03	7.40E-03
	Co-60		B-	1.68E-13	2.20E-13	3.32E-15	1.40E-15	2.90E-08		3.03E-02	2.90E-02
	Sr-82	Rb-82	EC	1.05E-13	1.00E-13	3.55E-12	4.20E-12	1.00E-08		6.97E-02	4.70E-02
	Y-90		B-	1.22E-19	1.00E-16	3.73E-12	3.10E-12	1.60E-09		3.77E-02	4.70E-02
	Cs-137	Ba-137m	B-	6.36E-14	5.60E-14	4.02E-13	1.20E-13	4.80E-09		4.20E-02	4.40E-02
	At-211	Po-212	A EC	4.65E-15	4.00E-15	6.42E-15	1.00E-15	1.10E-07		1.20E-04	6.30E-05
Other radionuclides	Cu-61		EC B+	8.90E-14	-	9.21E-13	-	1.20E-10	-	2.50E-02	-
	As-71		EC B+	5.51E-14	-	9.15E-14	-	5.00E-10	-	1.67E-02	-
	Se-72	As-72	EC	1.64E-13	-	7.20E-12	-	9.20E-10	9.20E-10	6.72E-02	-
	Nd-140	Pr140	EC	3.17E-15	-	2.11E-12 -	-	-	-	1.92E-02	-
	Tb-152		EC B+	1.26E-13	-	1.33E-12	-	-	-	9.98E-03	-
	Tb-155		EC	1.90E-14	-	8.06E-16	-	2.50E-10	-	7.36E-03	-
	Tb-156		EC	1.67E-13	-	3.09E-14	-	1.40E-09	-	2.27E-02	-
	Tb161		B-	1.41E-14	-	5.37E-15	-	1.20E-09	-	3.69E-02	-
	Tm-166		EC B+	1.61E-13	-	8.87E-14	-	2.80E-10	-	1.65E-02	-
	Yb-166	Tm-166	EC	1.70E-13	-	9.77E-14	-	1.19E-09	-	1.96E-02	-
	Tb-149		EC B+ A	1.17E-13	-	4.17E-13	-	3.10E-09	-	1.21E-02	-
	Bi-213	Po-213.Tl-209	EC B+ A	1.89E-13	-	2.20E-12	-	4.10E-08	-	4.55E-02	-

*The IAEA values for the different radionuclides are also listed (4)*.

**Table 5:** Results of the *A*_1_ and *A*_2_ values obtained with the MC method compared with the ones listed in the IAEA Safety guide (4).

		***A***_**1**_		***A***_**2**_	
	Radionuclide	**TBq**			
		**MCNPX**	**IAEA**	**MCNPX**	**IAEA**
Control group	Be-7	2.09E+01	2.00E+01	2.90E+00	2.00E+01
	Na-22	5.29E-01	5.00E-01	5.29E-01	5.00E-01
	Na-24	2.47E-01	2.00E-01	2.47E-01	2.00E-01
	Ca-47	5.16E-01	3.00E+00	3.54E-01	3.00E-01
	Co-58	9.89E-01	1.00E+00	9.89E-01	1.00E+00
	Co-60	5.95E-01	4.00E-01	5.95E-01	4.00E-01
	Sr-82	2.82E-01	2.00E-01	2.82E-01	2.00E-01
	Y-90	2.68E-01	3.00E-01	2.68E-01	3.00E-01
	Cs-137	1.57E+00	2.00E+00	6.66E-01	6.00E-01
	At-211	2.15E+01	2.00E+01	4.55E-01	5.00E-01
Other radionuclides	Cu-61	1.09E+00	1.00E-01	1.09E+00	2.00E-02
	As-71	1.82E+00		1.67E+00	
	Se-72	1.39E-01		1.39E-01	
	Nd-140	4.75E-01		4.75E-01	
	Tb-152	7.53E-01		7.53E-01	
	Tb-155	5.27E+00		3.81E+00	
	Tb-156	5.99E-01		5.99E-01	
	Tb161	7.11E+00		7.58E-01	
	Tm-166	6.23E-01		6.23E-01	
	Yb-166	5.88E-01		5.88E-01	
	Tb-149	8.56E-01	2.00E-01	8.56E-01	9.00E-05
	Bi-213	4.54E-01		4.54E-01	

#### 3.4.1. Results of the Control Group

**Figure 6:**
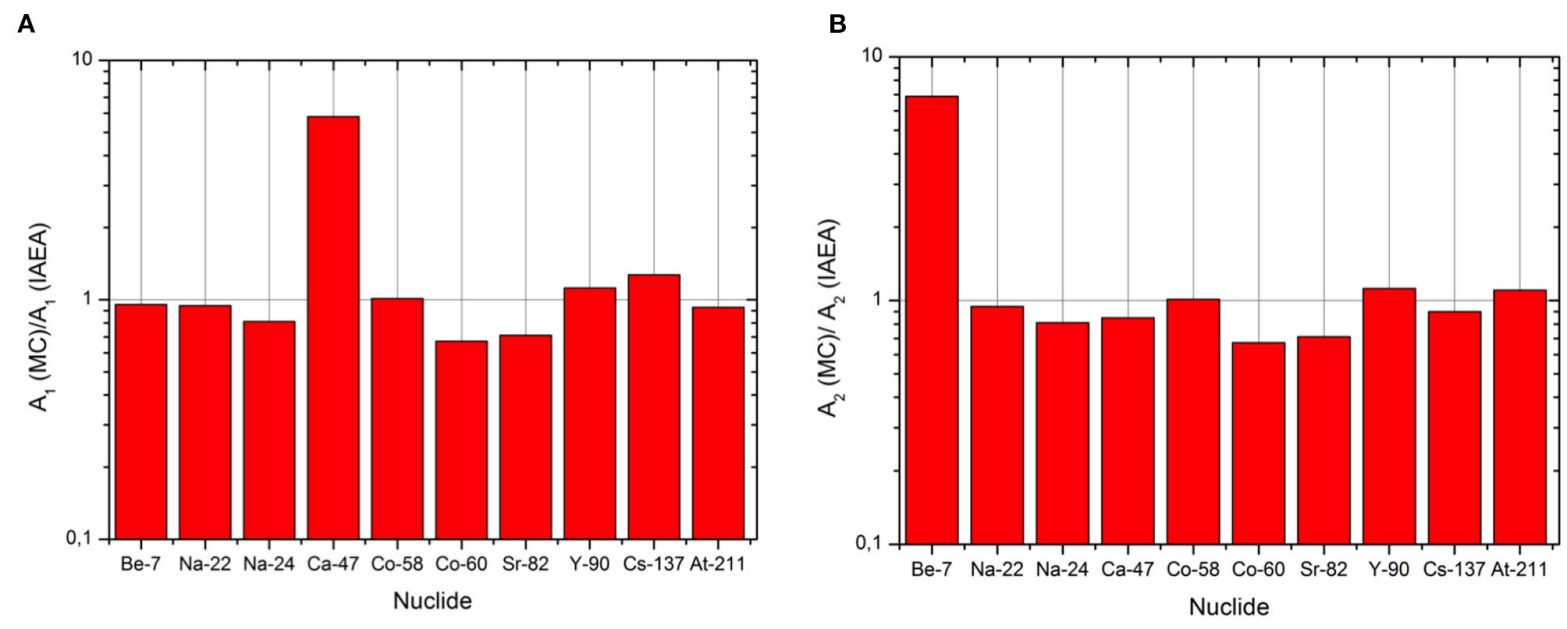
Ratio between the simulated values and the tabulated ones for *A*_1_ **(A)** and *A*_2_ **(B)** for the isotopes in the control group.

The first 10 cases represent what we called the *control group*, for which the IAEA values are available and tabulated. The two graphs in Figure 6 report the ratio between the MC simulated values and the IAEA tabulated. As we can observe, there is a good agreement between the results of the Monte Carlo simulations and the listed factors both in the calculation for *A*_1_ and *A*_2_ (the ratio is almost 1 in all the cases). There are two exceptions:

1. The exception for *A*_1_ is represented by the case of Ca-47 for which the recalculated value is smaller than the one in the Regulation. The explanation is found in the different ė_*b*_ dose coefficients. A reason for this discrepancy could be the use of different nuclear data sets for the beta decay of this radionuclide and the daughter included in the calculation (Sc-47).
2. An exception for *A*_2_ seems to be represented by the case of Be-7. As said previously, the *A*_2_ value is given by the minor of all the Q values. In the case of the Monte Carlo method, the limiting factor for the Be-7 is imposed by the *Q*_*D*_ value (2.90E+00 TBq), almost two orders of magnitude lower than the tabulated one (1.0E+03 TBq).
  Actually in the Regulation it is assumed that if *Q*_*D*_ results to be a value higher than 10^3^ TBq, *Q*_*D*_ shall be limited to 10^3^ TBq. Applying this rules, the *A*_2_ for Be-7 becomes limited by the gamma dose rate coefficient and equal to: 2.09 TBq.

The MC method is able to well reproduce the scenarios, the hypothesis and mostly the physics behind the Regulation. Moreover, those results allowed us to validate the MC simulation codes and apply them to obtain a dataset of *A*_1_ and *A*_2_ that have no value in IAEA tables and for which generic transport limits must be used. The relative errors of the simulations are always lower than 1% (statistical error) and not reported in the tables and the graph.

#### 3.4.2. Results and the Comparison for Electrons Emitters

**Figure 7:**
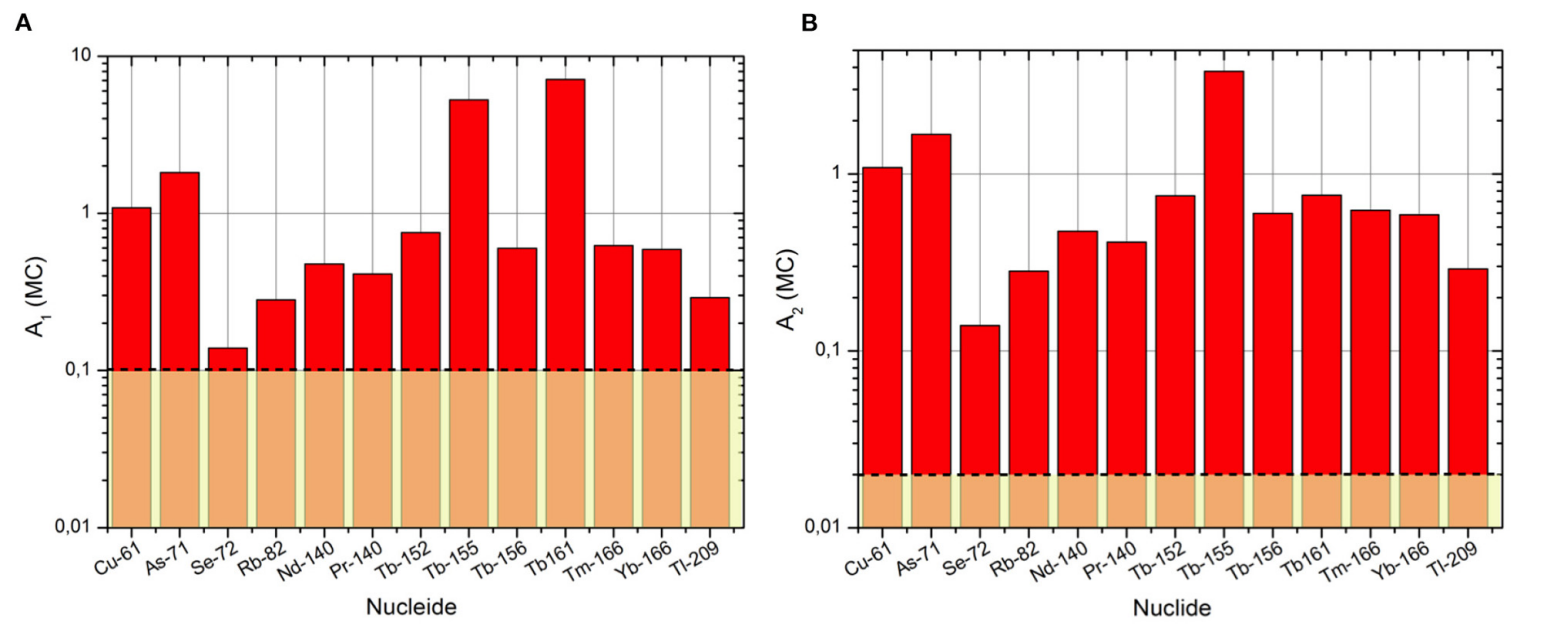
Simulated values with the Monte Carlo technique (MC) for *A*_1_ **(A)** and *A*_2_ **(B)** compared with the values of the Regulation (yellow rectangle) for the electron emitters.

The generic value imposed by the Regulation for beta emitters is 0,1 TBq for *A*_1_ and 0,02 TBq for *A*_2_ (Figure 7).

- In the case of the *A*_1_ values, we can observe that the Monte Carlo method does not involve a big increase of those limits. Among the cases examined, only for Cu-61, As-71, Tb-161 and Tb-155 an increase in the limit of one order of magnitude is observed, while in the remaining cases the increase is maximum of a factor 6.
- The gap between the regulatory values and the simulated ones is more evident in the case of the *A*_2_ data sets. In all the cases analyzed, in fact, the results of the MC method allow, an increase of the Transport limit of one or, in some cases (as for the Tb-155), two orders of magnitude.

#### 3.4.3. Results and the Comparison for Alpha Emitters

For the two alpha emitters with generic transport limits, Tb-149 and Bi-213, the A_1_/A_2_ the calculated values are respectively, 8.56E-01 TBq and 4.54E-01TBq. Applying the MC method we would observe that:

- the *A*_1_ limits are respectively, 2 and 4 times higher than the generic one (2.0E-01 TBq);
- the recalculated *A*_2_ values are four orders of magnitude higher than what is prescribed by the Regulation (9E-05 TBq)
- in the case of Tb-149 the limiting value is coming from the Q_*A*_, the dose from gamma source exposure.
- for Bi-213, instead, the lower of the Q values is the Q_*B*_, due to the beta dose to the skin.

#### 3.4.4. Comparison With Other Dataset

The values listed in the previous tables are also in good agreement with the ones obtained, for the same group of isotopes, from a working group of the Radiation Protection group at CERN. The main differences with the present study is the use of Fluka as the Monte Carlo software used for the calculations (18) and geometrical structures without a spherical symmetry. The basic principles of calculations remain the same. Some examples are reported in the Table 6. They are relative to the dose rate coefficients due to the beta particles ė_*b*_ and ḣ_*skin*_.

**Table 6:** Results of the ė_*b*_ and ḣ_*skin*_ dose coefficients from the Monte Carlo method with MCNPX and FLUKA.

**Isotope**	ė_*****b*****_ **[Sv** ***Bq***^**−1**^***h***^**−1**^**]**		ḣ_*****skin*****_ **[Sv** ***m***^**−2**^***TBq***^**−1**^***s***^**−1**^**]**	
	**MCNPX**	**FLUKA**	**MCNPX**	**FLUKA**
Co-60	3.32E-15	3.28E-15	3.03E-02	2.92E-02
Tb-149	4.17E-13	3.94E-13	1.21E-02	1.27E-02
Tm-166	8.87E-14	8.51E-14	1.65E-02	1.24E-02
Bi-213	2.2E-12	1.63E-12	8.42E-02	8.82E-02

## 4. Discussion

The development of new techniques of production of exotic radionuclides to use in systemic radiotherapy and imaging yields to the development of new containers to transport them. The radionuclides suitable for nuclear medicine purposes are characterized by short half-life. They are generally produced in nuclear reactors, cyclotrons or other accelerator facilities.

In the context of the transport the (short, few hours to few days) isotope's half life is an important factor: considering the time needed for transport from the point of production to the laboratories for the chemical saparation and the labeling (sometime those two are not in the same place) and then the transport of the final product to the hospital, the initial activity to be transported shall be much higher then the one actually used at the patients level.

Once the samples are irradiated, they shall undergo a series of chemical treatments before being coupled to biological substances to be injected in humans or animals for preclinical studies.

From the place of irradiation the samples containing the desired radionuclide is shipped to a chemical laboratory. The final product can be then used in the same place of production or it can be shipped again to other places like hospitals, imaging center or other research institutes.

Appropriate packages are needed to move the irradiated samples. In the first phase of this path the sample to transport is characterized by a high level of activity, generally due also to the presence of radioactive contaminants collected at the same time.

Due to the hours or days spent for the travel and the needs to take into account the decay of the radionuclides, the activities to transport suitable for the radiopharmaceutical production sometimes exceeds the values defined for the type A containers or industrial packages imposed by the IAEA. This higher hazard involves the use of more complex and safety demanding packages, called type B containers.

The value of activity to transport, different for each radionuclide, is the quantity defining the type of package to use for transport.

The International Atomic Energy Agency established a method, the so-called Q-system, based on different kind of exposures during an accident involving the damage of a transport container.

Those values are most of the time general and not based on specific calculations. Moreover, the nuclear data refers to not updated database and references to the used ones are difficult to identify.

The use of the Monte Carlo method for the evaluation of the transport limit *A*_1_ and *A*_2_ based on the Q-system as set by IAEA has been described. It has been used as a basis of an alternative method of calculation making use of Monte Carlo techniques and in particular of the software MCNPX to evaluate dose rate parameters in specific scenarios.

This method has been validated with a control group of nuclides with known/tabulated Q values. The results of the simulations, also in agreement with the ones obtained by other working groups, would allow an increase of the generic tabulated values. Among the analized cases we can cite the ones regarding two of the Terbium isotopes used in nuclear medicine: Tb-149 and Tb-161. For Tb-149, the recalculated values (*A*_1_ and *A*_2_: 8.56E-01 TBq) are two orders of magnitude higher then the one prescribed by the regulation (*A*_1_: 2.0E-01TBq, *A*_2_: 9.0E-05 TBq). While for Tb-161 applying the Monte Carlo method it would be possible to gain one order of magnitude for *A*_1_ (from 1.0E-01 extabilished from the regulation to 7.1E+00 TBq) and *A*_2_ (from 2.0E-02 extabilished from the regulation to 5.9E-01 TBq).

The increase of such limits would affect the choice of the type of transport package, allowing the use of more compact and cheaper containers, like type A. On the other hand it adds knowledge on the effective dose rate values, and then the hazard, associated to a single radionuclide, avoiding the use of generic common limits.

The strength of this method relies on the possibility to include in the calculations, all the phenomena and the effects linked to the particle interaction with matter.

A future development and improvement of these calculations must include Monte Carlo simulations to quantify the alpha emitter's hazard (for the *Q*_*C*_ evaluation) and a study of the dose due to the submersion accidental scenario (for the calculation of *Q*_*E*_) in case of gaseous sources. This may be done including in the simulations the information on the ICRP human phantom.

Additional study is needed also to better determine the Shielding Factor included in the *Q*_*B*_ calculations, the geometry and the material composing the shield associated to this calculation.

Recently an international working group managed by IAEA has been created with the aim of improve and update the Q-System method and databases (19). A new version of the Regulation for the transport of radioactive material including new limits will be published in the next years.

## Data Availability Statement

The raw data supporting the conclusions of this article will be made available by the authors, without undue reservation.

## Author Contributions

MM wrote the first draft of the manuscript. All authors contributed to the discussion concerning the results included in the manuscript and to its revision and read, approving the submitted version, and added comments to the discussion part, method presented and the results section.

## Funding

This research project has been supported by a Marie Sklodowska-Curie Innovative Training Network Fellowship of the European Commission's Horizon 2020 Program under contract number 642889, MEDICIS-PROMED. The Cyclotron Arronax was supported by CNRS, Inserm, INCa, the Nantes University, the Regional Council of Pays de la Loire, local authorities, the French government and the European Union. This work has been, in part, supported by a grant from the French National Agency for Research called Investissements d'Avenir, Equipex Arronax-Plus no ANR-11-EQPX-0004, Labex IRON no ANR-11-LABX-18-01 and ISITE NExT no ANR-16-IDEX-007.

## Conflict of Interest

SA was employed by Naogen Pharma. The remaining authors declare that the research was conducted in the absence of any commercial or financial relationships that could be construed as a potential conflict of interest.

## Publisher's Note

All claims expressed in this article are solely those of the authors and do not necessarily represent those of their affiliated organizations, or those of the publisher, the editors and the reviewers. Any product that may be evaluated in this article, or claim that may be made by its manufacturer, is not guaranteed or endorsed by the publisher.
